# Associations between cadmium exposure and neurocognitive test scores in a cross-sectional study of US adults

**DOI:** 10.1186/1476-069X-12-13

**Published:** 2013-02-05

**Authors:** Timothy Ciesielski, David C Bellinger, Joel Schwartz, Russ Hauser, Robert O Wright

**Affiliations:** 1 Institute for Quantitative Biomedical Sciences, Dartmouth College, Geisel School of Medicine at Dartmouth, Hanover, New Hampshire, USA; 2 Department of Environmental Health, Harvard School of Public Health, Boston, MA, USA; 3 Department of Neurology, Children’s Hospital, Boston, Boston, MA, USA; 4 Harvard Medical School, Boston, MA, USA; 5 Department of Preventive Medicine, Mount Sinai School of Medicine, New York, NY, USA

**Keywords:** Cadmium, Neurocognitive, Neuropsychological, NES2, NHANES, Attention, Smoking, Metals, Aging, Cognitive

## Abstract

**Background:**

Low-level environmental cadmium exposure and neurotoxicity has not been well studied in adults. Our goal was to evaluate associations between neurocognitive exam scores and a biomarker of cumulative cadmium exposure among adults in the Third National Health and Nutrition Examination Survey (NHANES III).

**Methods:**

NHANES III is a nationally representative cross-sectional survey of the U.S. population conducted between 1988 and 1994. We analyzed data from a subset of participants, age 20–59, who participated in a computer-based neurocognitive evaluation. There were four outcome measures: the Simple Reaction Time Test (SRTT: visual motor speed), the Symbol Digit Substitution Test (SDST: attention/perception), the Serial Digit Learning Test (SDLT) trials-to-criterion, and the SDLT total-error-score (SDLT-tests: learning recall/short-term memory). We fit multivariable-adjusted models to estimate associations between urinary cadmium concentrations and test scores.

**Results:**

5662 participants underwent neurocognitive screening, and 5572 (98%) of these had a urinary cadmium level available. Prior to multivariable-adjustment, higher urinary cadmium concentration was associated with worse performance in each of the 4 outcomes. After multivariable-adjustment most of these relationships were not significant, and age was the most influential variable in reducing the association magnitudes. However among never-smokers with no known occupational cadmium exposure the relationship between urinary cadmium and SDST score (attention/perception) was significant: a 1 μg/L increase in urinary cadmium corresponded to a 1.93% (95%CI: 0.05, 3.81) decrement in performance.

**Conclusions:**

These results suggest that higher cumulative cadmium exposure in adults may be related to subtly decreased performance in tasks requiring attention and perception, particularly among those adults whose cadmium exposure is primarily though diet (no smoking or work based cadmium exposure). This association was observed among exposure levels that have been considered to be without adverse effects and these levels are common in U.S. adults. Thus further research into the potential neurocognitive effects of cadmium exposure is warranted. Because cumulative cadmium exposure may mediate some of the effects of age and smoking on cognition, adjusting for these variables may result in the underestimation of associations with cumulative cadmium exposure. Prospective studies that include never-smokers and non-occupationally exposed individuals are needed to clarify these issues.

## Background

Cadmium is a heavy metal found in food, tobacco smoke, and certain occupational environments [1]. In adults, elevated cadmium exposure has been linked to a variety of neuropsychological deficiencies such as: reading difficulties, behavioral problems [2], poor visual-motor performance, complaints of decreased concentration [3], reduced attention, psychomotor speed, and memory [4] as well as lower cognitive scores among elderly adults with [5] or without concomitantly elevated zinc exposure [6]. In contrast, Nordberg et al. [7] found no association between blood cadmium and neurocognitive test scores in elderly adults. Three of these studies were done in occupational settings with small sample sizes, and the three larger studies only evaluated elderly adults. Thus there is a need for population-based studies of younger adults. In this study we analyzed a large representative sample of US adults ages 20–59, from the Third National Health and Nutrition Examination Survey (NHANES III) to determine if higher levels of urinary cadmium were associated with poorer performance in several tests from the Neurobehavioral Evaluation System 2 (NES2), a computer-based neurocognitive assessment [8,9].

In prior risk assessments kidney damage has been considered the most sensitive endpoint of cadmium toxicity and reference levels for urinary cadmium have been established to protect against this effect (EFSA 2009: 1 μg Cd/g creatinine, WHO/FAO 2011: 5.24 μg Cd/g creatinine) [1,10]. We compare our results to these reference levels to determine if any associations with adverse neurocognitive outcomes are present at exposure levels below these current thresholds of concern.

## Methods

### Data source and study population

NHANES III is a cross sectional survey of a representative sample of the United States population conducted between 1988 and 1994. It includes questionnaire data, medical examination data, and laboratory data from biological specimens [11,12]. Urinary cadmium information is publicly available for participants over 6 years of age, and neuropsychological screening was done on a random subsample of participants (n = 5662) between age 20 and 59 [13,14]. Additional information for NHANES III based analyses is available online: http://www.cdc.gov/nchs/nhanes/nh3data.htm[11]. The NHANES website (http://www.cdc.gov/nchs/nhanes/irba98.htm) notes that documented consent was obtained from participants and approval was obtained from the NHANES Institutional Review Board (its name changed to NCHS Research Ethics Review Board in 2003) [15].

### Exposure assessment

Cadmium accumulates in the kidney, and urinary cadmium concentration is considered a useful biomarker to estimate cumulative cadmium exposure [16]. We used urinary cadmium concentration assessed via atomic absorption spectrophotometry (AAS) with Zeeman background correction as the exposure metric for these analyses [17,18]. Because these were spot urine samples we included urinary creatinine concentration as an independent covariate in our models in order to account for individual variation in fluid intake/urine dilution [19].

### Covariates

Information on a number of potentially relevant covariates was available [11]. The covariates included in our multivariable-adjusted regression models were urinary creatinine (mg/dL), age (in years), sex, race-ethnicity (non-Hispanic white, non-Hispanic black, Mexican American, and other), smoking status (never, former, or current smoker), serum cotinine (μg/L), blood lead (μg/dL), language the exam was given in (Spanish or English), education (in years), and poverty income ratio (PIR - family income divided by the poverty threshold).

As discussed above, we included the independent variable for urinary creatinine in our models to control for differences in urine flow rate. This is a preferred method for avoiding potential biases related to differences in free water intake/urine dilution in epidemiologic studies that utilize urinary biomarkers of exposure [19]. The remainder of the variables in the models are known predictors of neurocognitive outcome [20-25], that 1) appear to have a relationship with cadmium exposure (data not shown) and 2) are not likely to be on the causal pathway between cadmium exposure and neurocognitive outcome. Thus we included them in the models to adjust for potential confounding. Note that there was not much difference between the urinary cadmium levels of males and females, consequently any potential confounding by this variable should be relatively small. However, the inclusion of this variable was crucial for the interaction analyses. There is evidence that cadmium may mimic sex hormones, and have different toxicokinetics in males and females [26], thus it was important to evaluate if the relationships between urine cadmium and the outcomes differed by sex.

Occupational information was available and was used to identify a subpopulation of never smokers who had no evidence of occupational cadmium exposure (details in the statistical analysis section). The secondary analyses in this subpopulation allowed us to evaluate associations with cadmium in the absence of potential confounding from unmeasured co-exposures present in tobacco smoke and certain work environments.

### Outcome measures

Neuropsychological performance was assessed with three subtests from the Neurobehavioral Evaluation System 2 (NES2), which is a computer-based screening program designed for epidemiologic studies [8]. Details on the use of NES2 in NHANES III have been previously published [21,27]. The first subtest was the Simple Reaction Time Test (SRTT). This subtest quantifies visual motor speed, and involves pushing a button when a specific shape appears on the screen. The second subtest was the Symbol Digit Substitution Test (SDST), which is a measure of attention and perception. In this subtest 9 digits are paired with 9 symbols in a grid, and the subject matches the correct digits with symbols on a second grid. The third subtest, the Serial Digit Learning Test (SDLT), is a measure of learning recall and short-term memory. Strings of 8 numbers are presented on the screen. The participant must then remember and enter the 8 digits. There are two summary measures for this subtest. The first summary measure is the number of trials required to meet the criterion. Criterion consists of doing the task correctly twice in a row (top coded at 8 trials max). Error scores are given for each trial (0 = 8 digits correct, 1 = 6–7 digits correct, and 2 = less than 6 digits correct) [13], and the second summary measure is the sum of these error scores. For each of these four outcome measures, lower scores correspond to better performance.

### Statistical analysis

For the SRTT and SDST analyses we used the GAMM function in R version 2.10.1 (The R Foundation for Statistical Computing, http://www.r-project.org/) to build mixed effect regression models. This approach accounts for clustering related to the hierarchical sampling structure of NHANES III by using a random intercept for each study location [28,29]. We specified weight variables in GAMM to appropriately account for unequal probabilities of selection created by the intentional oversampling of certain segments of the US population [12]. This approach was taken because standard survey statistics packages do not allow the use of penalized splines to assess nonlinearity. In order to consider potential non-linear relationships between urine cadmium and an outcome, or a covariate and an outcome, we initially modeled continuous predictor variables with penalized splines that were selected via a Generalized Cross Validation (GCV) procedure. GCV uses an automated iterative selection process to identify a smooth function which describes the relationship between two continuous variables [28,30].

The SRTT and SDST outcomes had roughly log normal distributions, and we log transformed these variables for the analyses. We constructed three models for each outcome. Model 1 included only urinary cadmium and an independent term for urinary creatinine. Model 2 added age, sex, race-ethnicity, reported smoking status, cotinine, blood lead, the language in which the exam was given (Spanish, English), education level, and Poverty Income Ratio. Model 3 was the same as model 2 except that the terms for smoking status and cotinine were removed. We performed this sensitivity analysis (model 3) because smoking is a source of cadmium exposure [1] and if any effect of smoking on cognition is mediated by cadmium, then adjusting for smoking may mask a cadmium effect. We initially included continuous variables in the models as splines, but if the GCV process did not provide evidence of non-linearity, we thereafter included this variable as a linear term. In the multivariable-adjusted SRTT and SDST models (model 2), GCV did not detect evidence of non-linearity, suggesting that urinary cadmium could be modeled as a linear term. For comparison, we modeled urinary cadmium as a linear term in models 1 and 3 as well. One participant had a very high urinary cadmium concentration (14.69 μg/L) that was over 18 standard deviations (0.77 μg/L) above the mean (0.64 μg/L), and was excluded from the regression models.

The two SDLT outcomes had discrete right censored distributions that were not transformable to a normal distribution. Therefore, we dichotomized these variables at the median and performed logistic regression analyses. We used *proc rlogist* in SAS callable SUDAAN (SAS 9.1.3 and 9.2: SAS Institute Cary, NC; SUDAAN 10.0.1 and 11.0.0: Research Triangle Institute Raleigh, NC), to build multivariable logistic regression models in the same manner as with the SRTT and SDST models, except that splines were not considered. The *proc rlogist* command in SUDANN allowed us to specify strata and primary sampling unit (PSU) variables to account for clustering related to the hierarchical sampling structure, and allowed us to specify weight variables to account for the oversampling of certain groups [12].

In order to determine which variables had the greatest influence on the urinary cadmium effect estimates in the multivariable-adjusted models, we re-ran model 2 several times for each of the 4 outcomes, leaving out one covariate each time. Upon removing the term for age from the SDST model, there was a convergence problem that resolved when urinary creatinine was modeled as a linear term. Therefore we modeled urinary creatinine with a linear term in the SDST confounding magnitude analyses. In order to investigate effect modification by sex, we added a sex-cadmium interaction term to model 2 for each of the four outcomes.

To further differentiate associations with cumulative cadmium exposure from associations with other aspects of aging we removed the continuous age variable from model 2 and added an ordinal age variable with 4 age categories (20–29, 30–39, 40–49, and 50–59 years). We then added an interaction term between urinary cadmium and this four category age variable to obtain stratum specific effect estimates for urinary cadmium within each of these age categories.

In order to evaluate associations with urinary cadmium in the absence of potential confounding from co-exposures in tobacco smoke and certain occupational environments we also evaluated models 1 and 2 (excluding the smoking status term) among never smokers who had no evidence of work-based cadmium exposure. Participants were considered to have potential occupational exposure if they reported a current or long held previous job in an industry that may involve cadmium exposure (mining, metal industries, chemicals/petroleum/coal products, rubber/plastics/leather products, electrical machinery/equipment/supplies) [31]. This resulted in the exclusion of 195 of the 2851 never smokers (6.8%) from these analyses.

## Results

A urinary cadmium level was available for 5572 (98%) of the 5662 NHANES III participants evaluated with the NES2 exam. Of these, SRTT data were available for 4848 (87%), and SDST data were available for 5021 (90%). For the SDLT, data on trials-to-criterion were available for 4845 (87%) and data on total-error-score were available for 4911 (88%). The median urinary cadmium concentration in the study population was 0.42 μg/L and the interquartile range was 0.19 - 0.82 μg/L (Table 1). Other characteristics of the study population are summarized in Table 1. For each of the 4 outcomes, people with worse scores tended to have higher urinary cadmium concentrations (Table 2).

**Table 1:** **Characteristics of the study population**^**a**^

	**N**	**(%)**		**N**	**Median**	**(IQR**^**b**^**)**
**Sex**			**Urinary Cadmium (μg/L)**	5572	0.42	(0.19 - 0.82)
Male	2548	(45.7)				
Female	3024	(54.3)	**Urinary Creatinine (mg/dL)**	5547	137	(79–197)
**Race-Ethnicity**			**Age (in years)**	5572	36	(27–45)
Non-Hispanic White	1900	(34.1)				
Non-Hispanic Black	1753	(31.5)	**Education (in years)**	5538	12	(10–14)
Mexican	1685	(30.2)				
Other Race	234	(4.2)	**Poverty Income Ratio**	5152	1.98	(1.02 - 3.36)
**Exam Given in Spanish**			**Serum Cotinine (μg/L)**	5269	0.57	(0.13 - 105)
No	4408	(86.6)				
Yes	681	(13.4)	**Blood Lead (μg/dL)**	5388	2.8	(1.6 - 4.6)
Missing	483					
**Smoking Status**			**NES2 Test Scores**			
Never Smoker	2851	(51.2)	**SRTT**	4848	229	(209–255)
Former Smoker	1039	(18.7)	**SDST**	5021	2.67	(2.29 - 3.20)
Current Smoker	1681	(30.2)	**SDLT**			
Missing	1		**trials-to-criterion**	4845	5	(3–8)
			**total-error-score**	4911	4	(2–10)

^a ^study population values, not weighted for oversampling.

^b ^interquartile range.

**Table 2:** **Urinary cadmium concentrations (μg/L) by neurocognitive test performance**^**ab**^

	**N**	**Median**	**Interquartile range**
**SRTT (in ms)**	4848 total		
**visual motor speed**			
158.5 - 209.3 (better)	1211	0.38	0.17 - 0.78
209.3 - 228.6	1213	0.40	0.19 - 0.80
228.6 - 254.6	1212	0.43	0.19 - 0.84
254.6 - 660.1 (worse)	1212	0.44	0.21 - 0.85
Missing	724	0.47	0.22 - 0.85
**SDST (in s/correct digit)**	5021 total		
**attention/perception**			
1.4 - 2.3 (better)	1223	0.31	0.14 - 0.59
2.3 - 2.7	1283	0.42	0.20 - 0.81
2.7 - 3.2	1254	0.45	0.21 - 0.85
3.2 - 22.2 (worse)	1261	0.52	0.22 - 1.03
Missing	551	0.45	0.21 - 0.85
**SDLT trials-to-criterion**	4845 total		
**learning recall/short-term memory**			
2 (better)	497	0.36	0.16 - 0.74
3 – 4	1624	0.37	0.17 - 0.72
5 – 7	1271	0.46	0.22 - 0.82
8 (worse)	1453	0.46	0.21 - 0.93
Missing	727	0.45	0.20 - 0.87
**SDLT total-error-score**	4911 total		
**learning recall/short-term memory**			
0 - 1 (better)	982	0.34	0.16 - 0.71
2 – 3	1066	0.38	0.18 - 0.68
4 – 9	1565	0.47	0.22 - 0.85
10 - 16 (worse)	1298	0.47	0.21 - 0.94
Missing	661	0.45	0.20 - 0.86

^a ^study population values, not weighted for oversampling.

^b ^urinary cadmium concentrations by quartiles of neurocognitive test performance.

### Simple reaction time test (SRTT: visual motor speed)

The effect estimate for urinary cadmium in the SRTT model adjusted only for urinary creatinine (model 1) was positive and significant (β_Cd_ = 0.0161, 95%CI: 0.0087, 0.0235) (Table 3). Due to the log transformation of the SRTT scores in these models, this effect estimate predicts a 1.61% higher SRTT score with each 1 μg/L increase in urinary Cd (higher scores indicate worse performance for each of these tests). However, after adjusting for age, sex, race-ethnicity, smoking status, cotinine, blood lead, language the exam was given in, education level, and Poverty Income Ratio (model 2) the effect estimate was reduced and no longer significant (β_Cd_ = −0.0034, 95%CI: -0.0122, 0.0054). In the sensitivity analysis which did not adjust for smoking status and cotinine (model 3), the urinary cadmium term remained non-significant (β_Cd_ = −0.0062, 95%CI: -0.0145, 0.0021). The terms for age and sex were most responsible for reducing the urinary cadmium effect estimate (data not shown). The multivariable-adjusted β for urinary cadmium was 0.0066 (95%CI: -0.0016, 0.0148) when not adjusting for age, and 0.0058 (95%CI: -0.0029, 0.0144) when not adjusting for sex.

**Table 3:** Associations between urinary cadmium concentration and neurocognitive test performance

**SRTT**				**SDST**		
**(visual motor speed)**				**(attention/perception)**		
			**% change in**			**% change in**
			**score with a**			**score with a**
			**1 μg/L increase**			**1 μg/L increase**
	**N**	**β**_**Cd**_^**a**^	**in Cd**^**b**^**(95%CI)**	**N**	**β**_**Cd**_^**a**^	**in Cd**^**b **^**(95%CI)**
**Model 1**	4833	0.0161*	1.61* (0.87, 2.35)	4998	0.0934*	9.34* (8.31, 10.37)
**Model 2**	4209	−0.0034	−0.34 (−1.22, 0.54)	4344	0.0068	0.68 (−0. 28, 1.64)
**Model 3**	4301	−0.0062	−0.62 (−1.45, 0.21)	4441	0.0095*	0.95* (0.03, 1.87)
**SDLT**						
**(learning recall/short-term memory)**						
**SDLT trials-to-criterion**				**SDLT total-error-score**		
	**N**	**OR**_**Cd**_^**c**^	**(95%CI)**	**N**	**OR**_**Cd**_^**c**^	**(95%CI)**
**Model 1**	4826	1.50*	(1.29 - 1.75)	4889	1.48*	(1.28 - 1.71)
**Model 2**	4202	1.03	(0.87 - 1.22)	4253	0.97	(0.77 - 1.22)
**Model 3**	4295	0.98	(0.83 - 1.16)	4348	0.97	(0.78 - 1.19)

Model 1. urinary cadmium and an independent term for urinary creatinine.

Model 2. add age, sex, race-ethnicity (non-Hispanic white, non-Hispanic black, Mexican American, and other), smoking status (never, former, or current smoker), serum cotinine, blood lead, language exam was given in (Spanish or English), education (in years), and poverty income ratio.

Model 3. same as model 2 but with smoking status and cotinine terms removed.

^a ^β for urinary cadmium (μg/L) with log transformed outcome.

^b ^percent change in test score associated with 1 μg/L increase in urinary cadmium (based on β). Note: higher scores correspond to worse performance.

^c ^OR for having a poor (above median) SDLT score associated with a 1 μg/L increase in urinary cadmium.

* significant at alpha = 0.05.

### Symbol digit substitution test (SDST: attention/perception)

The effect estimate for urinary cadmium in the SDST model adjusted only for creatinine was positive and significant (β_Cd_ = 0.0934, 95%CI: 0.0831, 0.1037) (Table 3). Due to the log transformation of SDST score in these models, this effect estimate predicts a 9.34% higher SDST score with each 1 μg/L increase in urinary Cd (again higher scores indicate worse performance). In the multivariable-adjusted model the effect estimate was reduced in magnitude and was no longer significant (β_Cd_ = 0.0068, 95%CI: -0.0028, 0.0164). However, in the sensitivity analysis, which did not adjust for smoking status or cotinine, the urinary cadmium term was significant (β_Cd_ = 0.0095, 95%CI: 0.0003, 0.0187). The term for age was most responsible for reducing the urinary cadmium effect estimate (data not shown). Due to the convergence issue discussed in the methods this cofounding assessment was made with urinary creatinine modeled as a linear term. When urinary creatinine was modeled as a linear term the effect estimate adjusted only for creatinine (model 1) was: β_Cd_ = 0.0934 (95%CI: 0.0831, 0.1037) the multivariable-adjusted effect estimate (model 2) was: β_Cd_ = 0.0082 (95%CI: -0.0014, 0.0178), and the multivariable-adjusted effect estimate not adjusted for age was: β_Cd_ = 0.0638 (95%CI: 0.0536, 0.0739).

### Serial digit learning test (SDLT: learning recall/short-term memory)

In the SDLT trials-to-criterion model adjusted only for creatinine, the odds of a having a poor score increased as urinary cadmium level increased (Table 3). In this model, the Odds Ratio (OR) for having a poor (above median) trials-to-criterion score associated with a 1 μg/L increase in urinary cadmium was 1.50 (95%CI: 1.29-1.75). This association was no longer present after multivariable-adjustment (OR_Cd_ = 1.03, 95%CI: 0.87-1.22) even when smoking status and cotinine were removed from the model (OR_Cd_ = 0.98, 95%CI: 0.83-1.16). The age term was most responsible for reducing the urinary cadmium effect estimate (data not shown). When not adjusting for age the multivariable-adjusted OR_Cd_ was 1.37 (95%CI: 1.15-1.63). The pattern of results was similar in the SDLT total-error-score analysis (Table 3, and when not adjusting for age the multivariable-adjusted OR_Cd_ was 1.28, 95%CI: 1.08-1.52).

### Sex interaction analyses

None of the sex-cadmium interaction terms were significant for any of the outcomes.

### Cadmium associations within four age categories

Stratum specific effect estimates for urinary cadmium within each of four age categories (20–29, 30–39, 40–49, and 50–59 years) are presented in Table 4. In this analysis the multivariable effect estimate for SDST (attention/perception), which was not significant across the full age spectrum, was larger in magnitude and significant in 3 of the 4 age categories.

**Table 4:** Associations between urinary cadmium concentration and neurocognitive test performance by age category

**SRTT**			**SDST**	
**(visual motor speed)**			**(attention/perception)**	
		**% change in**		**% change in**
		**score with a**		**score with a**
		**1 μg/L increase**		**1 μg/L increase**
	**β**_**Cd**_^**a**^	**in Cd**^**b **^**(95%CI)**	**β**_**Cd**_^**a**^	**in Cd**^**b **^**(95%CI)**
**20-29 yrs.**	0.0132	1.32 (−1.02, 3.65)	0.0289*	2.89* (0.36, 5.42)
**30-39 yrs.**	−0.0059	−0.59 (−2.15, 0.97)	−0.0030	−0.30 (−2.02, 1.42)
**40-49 yrs.**	−0.0012	−0.12 (−1.33, 1.09)	0.0161*	1.61* (0.26, 2.95)
**50-59 yrs.**	−0.0046	−0.46 (−1.79, 0.88)	0.0196*	1.96* (0.46, 3.47)
**SDLT**				
**(learning recall/short-term memory)**				
**SDLT trials-to-criterion**			**SDLT total-error-score**	
	**OR**_**Cd**_^**c**^	**(95%CI)**	**OR**_**Cd**_^**c**^	**(95%CI)**
**20-29 yrs.**	0.89	(0.61, 1.30)	1.02	(0.66, 1.58)
**30-39 yrs.**	1.11	(0.74, 1.66)	0.96	(0.67, 1.37)
**40-49 yrs.**	1.21	(0.95, 1.55)	1.17	(0.91, 1.52)
**50-59 yrs.**	0.89	(0.70, 1.15)	0.83	(0.63, 1.08)

Model adjusted for urinary cadmium, urinary creatinine, sex, race-ethnicity (non-Hispanic white, non-Hispanic black, Mexican American, and other), smoking status (never, former, or current smoker), serum cotinine, blood lead, language exam was given in (Spanish or English), education (in years), and poverty income ratio.

^a ^β for urinary cadmium (μg/L) with log transformed outcome.

^b ^percent change in test score associated with 1 μg/L increase in urinary cadmium (based on β). Note: higher scores correspond to worse performance.

^c ^OR for having a poor (above median) SDLT score associated with a 1 μg/L increase in urinary cadmium.

* significant at alpha = 0.05.

### Cadmium associations among never smokers with no known occupational exposure

In analyses restricted to never smokers with no evidence of work-based cadmium exposure (Table 5) the results were roughly similar with two notable exceptions: 1) the multivariable adjusted effect estimate in the SDST analysis (attention/perception) was larger in magnitude and significant (β_Cd_ = 0.0193, 95%CI: 0.0005, 0.0381), and 2) the multivariable adjusted effect estimate in the SDLT total-error-score analysis (learning recall/short-term memory) was larger in magnitude and borderline significant (OR_Cd_ = 1.45, 95%CI: 0.99 - 2.14).

**Table 5:** Associations between urinary cadmium concentration and neurocognitive test performance among never smokers with no known occupational exposure

**SRTT**				**SDST**		
**(visual motor speed)**				**(attention/perception)**		
			**% change in**			**% change in**
			**score with a**			**score with a**
			**1 μg/L increase**			**1 μg/L increase**
	**N**	**β**_**Cd**_^**a**^	**in Cd**^**b **^**(95%CI)**	**N**	**β**_**Cd**_^**a**^	**in Cd**^**b **^**(95%CI)**
**Model 1**	2286	0.0299*	2.99* (1.39, 4.59)	2371	0.0922*	9.22* (6.99, 11.44)
**Model 2**	1986	−0.0088	−0.88 (−2.69, 0.93)	2050	0.0193*	1.93* (0.05, 3.81)
**SDLT**						
**(learning recall/short-term memory)**						
**SDLT trials-to-criterion**				**SDLT total-error-score**		
	**N**	**OR**_**Cd**_^**c**^	**(95%CI)**	**N**	**OR**_**Cd**_^**c**^	**(95%CI)**
**Model 1**	2270	1.70	(0.99 – 2.93)	2301	2.25*	(1.67 – 3.05)
**Model 2**	1967	1.12	(0.82 - 1.53)	1991	1.45	(0.99 – 2.14)

Model 1. urinary cadmium and an independent term for urinary creatinine.

Model 2. add age, sex, race-ethnicity (non-Hispanic white, non-Hispanic black, Mexican American, and other), serum cotinine, blood lead, language exam was given in (Spanish or English), education (in years), and poverty income ratio.

^a ^β for urinary cadmium (μg/L) with log transformed outcome.

^b ^percent change in test score associated with 1 μg/L increase in urinary cadmium (based on β). Note: higher scores correspond to worse performance.

^c ^OR for having a poor (above median) SDLT score associated with a 1 μg/L increase in urinary cadmium.

* significant at alpha = 0.05.

## Discussion

### Interpretations

In this study of U.S. adults age 20–59 years, higher levels of urinary cadmium were associated with worse performance on each of four neuropsychological tests when the analyses were adjusted only for urinary creatinine. These relationships were not significant after adjustment for the full set of potential confounding variables (model 2). Adjustment for age in particular mitigated the effect estimates for urinary cadmium. However, the relationship between higher urinary cadmium levels and poor SDST scores was significant in the multivariable-adjusted model that was not adjusted for smoking variables (model 3). Smoking is a source of cadmium exposure [1] and smoking has been associated with neurocognitive deficiencies [20]. Therefore cadmium may mediate some of the effect of smoking on neurocognitive performance. If so, including smoking variables in the models could underestimate the magnitude of associations with cadmium, because cadmium-independent and cadmium-dependent effects of smoking would be difficult to disentangle.

To further explore this issue we performed analyses restricted to never smokers with no evidence of occupational cadmium exposure. These exclusions removed potentially confounding co-exposures in tobacco smoke and certain occupational environments from the analyses, thus allowing us to evaluate associations that should be related primarily to dietary cadmium exposure [1,20,31,32]. In these restricted analyses we found that the association between higher urinary cadmium and worse SDST score was still significant after multivariable adjustment (β_Cd_ = 0.0193, 95%CI: 0.0005, 0.0381). Because the SDST assesses attention and perception, this data suggests that elevated cadmium exposure in adults may be associated with reduced capacity in these cognitive functions.

The effect estimate for urinary cadmium among never smokers without occupational exposure (β_Cd_ = 0.0193) predicts a 1.93% (95%CI: 0.05, 3.81) increase in SDST score for a 1 μg/L increase in urinary cadmium. This suggests that moving from the 25th to the 75th percentile of urinary cadmium (0.19 to 0.82 μg/L) would correspond to 1.22% increase in SDST score. Higher scores indicate worse performance. While this effect size is small in magnitude, low-level cadmium exposure is nearly ubiquitous [10]. Thus if this association reflects a causal relationship, there may be a public health impact [33]. Furthermore, as we discuss below, this effect size and it’s public health impact may be underestimated due to the adjustment for age.

We also note here that the fully adjusted effect estimate for SDLT total error score was borderline significant when restricting to never smokers with no known occupational cadmium exposure (OR_Cd_ = 1.45, 95%CI: 0.99 - 2.14). This provides evidence for a potential association with decreased performance in learning recall and short-term memory as well.

For the 4 outcomes evaluated, adjusting for age produced the largest change in the urinary cadmium effect estimates, and in each case, age adjustment resulted in a decrease in the effect estimate. Because urinary cadmium concentration is a marker of cumulative exposure it tends to increase with age (bivariate model with log transformed urinary cadmium: β_age_ = 0.0262, 95%CI: 0.0227, 0.0296). Cognitive performance on each of the four tests decreased with age in our study population and this is consistent with the findings of Kreig et. al. in 2001 [21] (bivariate models: SRTT β_age_ = 0.0012 [95%CI: 0.0008, 0.0017]; SDST β_age_ = 0.0097 [95%CI: 0.0091, 0.0102]; OR for having a poor [above median] SDLT score associated with a 1 year increase in age: SDLT trials-to-criterion: 1.04, 95%CI: 1.03-1.05, SDLT total-error-score: 1.03, 95%CI: 1.02-1.04). The relationship between age and urinary cadmium concentration makes it difficult to determine if a portion of the age-associated cognitive changes may be due to cumulative cadmium exposure (i.e. cadmium may be on the causal pathway between age and neurocognitive performance). For this reason, adjusting for age may result in the underestimation of cadmium-test score associations. We nevertheless observed a significant association between urinary cadmium concentration and SDST scores despite adjusting for age (in the analysis restricted to never smokers with no known occupational exposure), and thus the association could be larger in magnitude than reported here. Though there is some instability in the urinary cadmium effect estimates within age strata in the SDST analysis (learning recall/short-term memory), these results (Table 4) are consistent with interpretation that adjusting for age may result in the underestimation of the urinary cadmium-SDST association.

With respect to visual motor speed (SRTT) the age interaction analysis is intriguing. Interestingly, a trend of worse performance with higher urinary cadmium was observed only in the youngest age group (20–29 years of age), suggesting that age modifies the relationship between cadmium and visual motor speed. The direction of this effect estimate suggests that young adults may be vulnerable to an adverse effect of cadmium on visual motor speed, perhaps because their baseline performance speed is slightly faster than the older age groups.

None of the cadmium-sex interaction terms were significant, but future studies may still benefit from considering cadmium-sex interactions, in light of the evidence that cadmium may mimic sex hormones, and have different toxicokinetics in males and females [26].

### Comparison to previous findings

As described in the introduction, there are a limited number of epidemiologic studies which looked specifically for associations between cadmium exposure and neurocognitive outcomes in adults. One study in Navy recruits (n = 40) found that high hair cadmium levels were significantly correlated with lower reading levels and behavioral problems but the analysis did not consider potential confounding [2]. Another small study (n = 31) evaluated occupationally exposed workers from a refrigerator coil manufacturing plant and found that workers with higher urinary cadmium levels had worse performance in tests of attention/psychomotor speed, and memory [4]. However, an alternate analysis found no significant relationships between urinary cadmium and performance after accounting for age and education. In a study of 89 workers (42 exposed and 47 control), urinary cadmium was significantly associated with poor visuomotor performance (symbol digit substitution and simple reaction time tests), even after adjusting for age, alcohol, exposure to other neurotoxicants, neuroactive medications, and years of schooling [3]. Case control studies suggest that elevated cadmium exposure or differences in cadmium processing may be associated with violent criminal behavior [34] and dementia/Alzheimer’s disease [35,36], but the results have been inconsistent [37,38].

In addition to these small studies, three population-based studies have been reported in elderly adults. One of these studies involved the evaluation of trace minerals in drinking water and dementia/cognitive screening of elderly people in China (n =1,016) [5]. This study did not detect an association between cognitive score and water cadmium levels but did identify a significant zinc-cadmium interaction. Participants with high levels of both zinc and cadmium in their water had lower cognitive scores. This group published another population-based study of elderly people in China that evaluated metal biomarkers in blood plasma rather than in drinking water (base study population: n = 2000, blood sample subpopulation: n = 188) [6]. Here the authors reported an association between higher plasma cadmium levels and lower composite cognitive scores after adjusting for age, gender, education, APOE genotype and BMI. A third study of elderly people was conducted in Stockholm (study population: n = 804, subsample analyzed for blood cadmium: n = 763) [7]. In this study no association was found between blood cadmium and mini-mental status exam (MMSE) scores, but in this report the composition of their regression models is unclear, and thus it is difficult to assess how potential confounding was addressed.

The previous work most comparable to our own in terms of outcomes measures (NES computerized neurobehavioral testing) is the study by Viaene et al [3]. The primary difference in the Viene et. al. study is that it was done in an occupational study population with higher exposure levels. In this work the authors found significant relationships between urinary cadmium and poor performance in both a simple reaction time test (visual motor speed) and a symbol digit substitution test (attention and perception). The results of our unadjusted SRTT analyses were consistent with their findings, although we did not detect a significant association between urinary cadmium and SRTT after multivariable adjustment. We found a similar relationship between urinary cadmium and SDST scores, and this association was significant when we made our models more similar to those of Viaene et al., by not adjusting for smoking. In our study this relationship with SDST was also significant in the subpopulation of never smokers with no known occupational cadmium exposure. Our study primarily involved low-level non-occupational cadmium exposures, and we controlled for different (as well as more) potential confounding variables. These factors may explain the differences in the findings of the studies.

The other study which assessed similar neurocognitive testing outcomes (Hart et. al 1989) involved the evaluation of 31 occupationally exposed workers, and the authors reported decreased attention, psychomotor speed, and memory in workers with higher urinary cadmium levels [4]. Though these associations were no longer significant after adjusting for age and education, the similarity of identified domains is worth noting.

The exposure metrics, exposure sources, exposure levels, sample sizes, age of participants, ethnicity of participants, outcome metrics, and consideration of potential confounders vary greatly among the prior epidemiologic studies. Despite these differences, 3 of the 4 large population-based studies, including our own, provide evidence linking cadmium exposure to worse neurocognitive performance in adults [5-7]. Additionally, the studies which attempted to evaluate neurocognitive domains, including our own, have identified similar domains: attention/perception (and perhaps memory) [3,4]. Furthermore, there is evidence that cadmium exposure may be associated with adverse neurodevelopmental outcomes in children (briefly reviewed in [39]).

With respect to possible mechanisms, laboratory animal experiments have demonstrated that cadmium exposure can affect neurotransmitter function, electrophysiological parameters, and behavior [40-47]. Combined with the epidemiologic studies, these reports from experimental toxicology increase the public health concerns about cadmium exposure and neurocognition.

### Implications for cadmium risk assessments

Recent risk assessments have established urinary cadmium reference levels to protect against kidney damage (EFSA 2009: 1 μg Cd/g creatinine, WHO/FAO 2011: 5.24 μg Cd/g creatinine), as this has been considered the most sensitive endpoint of cadmium toxicity [1,10]. In our study we did not use creatinine-standardized urinary cadmium to assess exposure. We instead included urinary creatinine as an independent term in our regression models as recommended by Barr et al. 2005 [19], but we can compare our results to these reference levels by evaluating our models within the exposure ranges defined by these reference levels. We added a reference level-cadmium interaction term to our fully adjusted SDST models and extrapolated stratum specific effect estimates for urinary cadmium among participants with 1) less 1 μg Cd/g creatinine, 2) 1–5.24 μg Cd/g creatinine, and 3) greater than 5.24 μg Cd/g creatinine. Excluding the extreme outlier there were only 2 participants with urinary cadmium levels above 5.24 μg Cd/g creatinine in the full study population, and among the never smokers with no known occupational cadmium exposure there were no urinary cadmium concentrations above this level, thus the highest exposure category could not be evaluated in these analyses.

In the general study population we found evidence that the inverse relationship between urinary cadmium concentration and SDST performance may be present among those with 1–5.24 μg Cd/g creatinine (β_Cd_ = 0.0114, 95%CI: -0.0016, 0.0244) but not present among those with less 1 μg Cd/g creatinine (β_Cd_ = −0.0133, 95%CI: -0.0298, 0.0031). Among the never smokers with no known occupational cadmium exposure we saw a similar pattern, however the association magnitude was much greater and highly significant among those with 1–5.24 μg Cd/g creatinine (1–5.24 μg Cd/g creatinine: β_Cd_ = 0.0510, 95%CI: 0.0217, 0.0804, less than 1 μg Cd/g creatinine: β_Cd_ = −0.0223, 95%CI: -0.0509, 0.0064). If this relationship reflects a casual mechanism then our data suggests that neurocognitive performance may be a sensitive endpoint of cadmium toxicity in adults and that the WHO/FAO reference level may not protect against this effect.

Recent evidence suggests that decreased rates of smoking are contributing to decreases in cadmium exposure in the U.S [48]. However, our study suggests that non-smoke, non-occupation based cadmium exposure may be associated with adverse neurocognitive outcomes in adults at levels below 5.24 μg Cd/g creatinine (the current WHO/FAO reference level). If this association reflects a causal relationship then common low level dietary cadmium exposures may be responsible decreased neurocognitive function in many U.S. adults. Further neurocognitive research should be conducted and new risk assessments for cadmium may be needed. It is important to note that associations with adverse neurodevelopmental outcomes in children have been recently been reported at cadmium levels below 1 μg Cd/g creatinine [39] and therefore the findings in children may have a larger impact on future risk assessments.

### Limitations and strengths

Our study is limited by the cross-sectional nature of the data, which restricts our ability to assess the temporal relationships between variables. However the long half-life of cadmium in the body (> 10 years) and the cumulative nature of the urinary cadmium exposure metric [16] suggest that at least some component of the exposure measurement reflects cadmium exposure which occurred well before the neurocognitive outcome measurement. We also note that our findings, like those of any observational study, may reflect non-causal relationships related to uncontrolled confounding.

The main strengths of our analysis include: a large sample size, adjustment for multiple potential confounders, sufficient information on exposure routes to evaluate nonsmoking nonoccupational cadmium exposure, and the use of an objective computer-based neuropsychological evaluation that was designed for epidemiologic applications [9]. Additional strengths of our analysis include use of penalized splines to assess and account for nonlinear relationships, as well as the use of a dataset that was designed to be representative of the U.S. population.

## Conclusions

Overall, these results provide support for the evidence suggesting that cadmium exposure may be associated with diminished neurocognitive performance in adults. The relationships observed in this study were detected at cadmium exposure levels that are 1) typical of US adults and 2) below the current WHO/FAO reference level. The associations between urinary cadmium and neurocognitive outcomes prior to multivariable-adjustment may be due in part to confounding by age. However the multivariable-adjusted models and age stratified analyses indicate that confounding by age does not completely explain the relationship between urinary cadmium and SDST scores, a measure of attention and perception. Confounding by co-exposures in tobacco smoke and occupational environments also cannot explain this association, as this relationship was significant among never smokers with no known work based cadmium exposure (dietary cadmium is the primary source of exposure in this subpopulation). These findings combined with evidence from laboratory experiments and prior epidemiologic studies highlight the need for population based prospective studies to evaluate the potential neurotoxic effects of cadmium exposure.

## Abbreviations

Cd: Cadmium; CDC: Centers for Disease Control and Prevention; GCV: Generalized Cross Validation; NCHS: National Center for Health Statistics; NES2: Neurobehavioral Evaluation System 2; NHANES III: Third National Health and Nutrition Examination Survey; OR: Odds Ratio.

## Competing interests

One of the authors (David Bellinger), and a person listed in the acknowledgements (Antonella Zanobetti) are on the EH editorial staff (also Philippe Grandjean and Katherine Herz are members of the HSPH EH department, and I have TA’d a course for David Wegman at HSPH), but we are not aware of any actual or potential competing interests.

## Authors’ contributions

TC performed the statistical analyses with the help of feedback from the other authors. DB, JS, RH, and RW served on the dissertation committee for TC and as this work was part of TCs dissertation, the analytic approach and results were discussed at dissertation committee meetings. TC wrote the manuscript draft. The authors provided comments/edits to the manuscript, and approved the final manuscript. RW was the senior author.
